# Isolation of *Plasmodium falciparum* by flow-cytometry: implications for single-trophozoite genotyping and parasite DNA purification for whole-genome high-throughput sequencing of archival samples

**DOI:** 10.1186/1475-2875-11-163

**Published:** 2012-05-14

**Authors:** Anne Boissière, Céline Arnathau, Christophe Duperray, Laurence Berry, Laurence Lachaud, François Renaud, Patrick Durand, Franck Prugnolle

**Affiliations:** 1 Laboratoire MIVEGEC, UMR IRD 224 CNRS 5290 UMI, 911 Av. Agropolis, 34394, Montpellier, Cedex 5, France; 2 Montpellier Rio Imaging, U847 IRB, 27 avenue du Professeur Grasset, Montpellier, France; 3 Laboratoire DIMP, CNRS UMR5235, Université de Montpellier 2, Place Eugene Bataillon, 34095, Montpellier, France

**Keywords:** *Plasmodium*, FACS, Cytometry, Genotyping, Whole genome sequencing

## Abstract

**Background:**

Flow cytometry and cell sorting are powerful tools enabling the selection of particular cell types within heterogeneous cell mixtures. These techniques, combined with whole genome amplification that non-specifically amplify small amounts of starting DNA, offer exciting new opportunities for the study of malaria genetics. Among them, two are tested in this paper: (1) single cell genotyping and (2) parasite DNA purification for subsequent whole genome sequencing using shotgun technologies.

**Methods:**

The method described allows isolation of *Plasmodium falciparum* trophozoites, genotyping and whole genome sequencing from the blood of infected patients. For trophozoite isolation, parasite and host nuclei are stained using propidium iodide (PI) followed by flow cytometry and cell sorting to separate trophozoites from host cells. Before genotyping or sequencing, whole genome amplification is used to increase the amount of DNA within sorted samples. The method has been specifically designed to deal with frozen blood samples.

**Results and conclusion:**

The results demonstrate that single trophozoite genotyping is possible and that cell sorting can be successfully applied to reduce the contaminating host DNA for subsequent whole genome sequencing of parasites extracted from infected blood samples.

## Background

Flow cytometry and cell sorting are powerful technologies that allow the physical separation of a cell, or a particle of interest, from a heterogeneous cell mixture. These techniques, combined with specific technologies in genetics such as whole genome amplification, provide new opportunities for malaria genetic studies. Among them two are explored in this article: (i) single trophozoite genotyping and (ii) parasite DNA purification for subsequent next generation shotgun sequencing.

### Single cell genotyping

Hosts are often infected with more than one genotype of the same pathogen. Multiclonal infections arise from infections with a genetically diverse inoculum or from re-infection before an existing infection is cleared [1]. Ecological interactions between these lineages within hosts can influence the parasite’s life history traits (virulence, transmission) and can thus have important consequences for their epidemiology and evolution [2,3].

Despite their ecological and evolutionary importance, mixed infections are frequently overlooked in natural conditions. This is true for malaria parasites, where most population genetic studies are performed using total DNA extracted from blood samples of infected patients. In areas of low transmission, most patients are infected with only one haploid parasite (one haplotype) whereas in areas of high transmission, a large proportion of patients carry multiple haplotypes [4]. Although both single and mixed-infections can be used to globally estimate allele frequencies at each locus [5], it remains difficult to assess the combinations of alleles at different loci. This information can yet be fundamental for understanding the population dynamics, evolution and biology of the parasite. Several methods have been proposed to circumvent this problem. One is to adapt the patient sample to culture and then dilute it to create monoclonal cultures [6]. However it is only possible to apply this method to very fresh, or cryopreserved, samples and it is very labour intensive. In addition this method may select for culturable variants. A second possibility is to use statistical methods to predict putative haplotypes. Several methods exist but, practically, they are difficult to apply to mixed samples containing more than two malaria strains [7,8]. As a consequence, when the allele combinations at different loci are the point of interest, mixed infections are often discarded to avoid analytical problems intrinsic to the handling of mixed genotypes.

One possibility to overcome these problems associated with multiple infections is to work directly at the scale of one parasite (one genome) and to genotype individual parasites contained in a patient’s blood. Such methods, unfathomable a couple of years ago are now possible thanks to the development and improvement of different tools. These include: i) efficient means to isolate and sort single cells, such as fluorescence-activated cell sorting [9], or laser capture micro-dissection; and ii) whole genome amplification (WGA) tools which allow the generation of a high quantity of genomic DNA from a very small initial quantity. Until now, single-cell sorting and genotyping have not been tested with malaria parasites.

### *Plasmodium falciparum* DNA enrichment and whole genome sequencing

Another area of interest of flow cytometry and cell sorting for the study of malaria genetics is the separation of parasite cells from those of the host for subsequent whole genome sequencing of the parasite.

One major problem to sequencing malaria infected blood samples using shotgun technologies is the presence of “contaminating” host DNA [10]. Because of the large size discrepancy between the human and *Plasmodium* genome, the presence of even a small number of host cells compared to parasite cells may preclude to obtain good sequence coverage of the parasite’s genome at a reasonable cost. Methods that enrich DNA samples with parasite DNA, or that remove host nucleated cells from samples, are imperative for efficient sequencing of the parasite genome [11,12].

Several methods have been proposed to purify the parasite DNA before shotgun sequencing. Sustained *in vitro* culture of the parasite in a human DNA free medium is one possibility. This method nevertheless demands a lot of time and expertise and is currently limited to species that can be adapted in culture. Another possibility is to selectively remove the leucocytes from the patient’s blood. Several methods have been proposed with various degrees of efficiency but they all require samples for which cytoplasmic membrane integrity is preserved (i.e. very fresh samples or cryopreserved samples in medium that maintains membrane integrity) [10]. Finally, a method based on hybrid selection of the targeted genome has been proposed. Its principle is to enrich the parasite DNA from a DNA mixture by specifically hybridizing it on special baits [11]. This technique can be applied to any DNA extract obtained from fresh or frozen archived samples at a modest cost. One limitation of this method however is that it can only be applied to pathogens for which complete genome sequences are known, or those of very closely related species. In addition, it is likely that regions of the genome displaying high levels of polymorphism (such as regions coding for antigenic proteins) could be underrepresented in the final sequence due to low affinity with baits during hybridization.

Again flow cytometry and cell sorting can provide a good alternative to these methods for several reasons: (i) they can be applied to any *Plasmodium* species infecting any mammal host, as long as it is possible to distinguish the parasite populations from the host nucleated blood cell population, (ii) they can be applied to any kind of blood samples, fresh or frozen, as long as enough host and trophozoite nuclei (which have very different sizes) remain intact for sorting and separation.

In this paper, flow cytometry and cell sorting technologies were tested for these two applications. The aim was to isolate trophozoites using fluorescence-activated cell sorting from the blood of infected patients, and genotype or sequence them, following whole genome amplification. The present protocol was developed from previously published protocols and was specifically designed to work on archived infected blood samples that have been conserved frozen.

## Methods

### General method of isolation and applications

The detailed protocol outlines trophozoite cell preparation from archived frozen samples (conserved at minus 20°C), characterization and cell isolation. It is followed by whole-genome amplification that provides a template for single-cell microsatellite genotyping and multiple-cell whole-genome sequencing. The protocol breaks down into five separate steps. (1) Preparation of trophozoite suspension from frozen infected venous blood; (2) Characterization of nucleus-stained trophozoites by flow cytometry; (3) Isolation of trophozoites using an automatic cell sorter device; (4) Trophozoite whole genome amplification; (5) Applications: a- single-trophozoite microsatellite genotyping or, b- whole genome sequencing.

### Preparation of trophozoite suspension from frozen blood samples

To optimize the following protocol, several protocols described in previous studies [13-16] were combined. Trophozoite isolation was carried out using a 40 μL sample of *Plasmodium*-infected venous blood added to 200 μL of NH4Cl-KHCO3 buffer (0.15 M NH4Cl, 1 g/L KHCO3, Ratio 1:5 v/v), which induces the bursting of red blood cells. The mixture was kept on ice for 5 min. Following centrifugation at 15,129xg for 5 min at 4°C, the pellet was washed twice by re-suspension in 200 μL of Buffer I (15 mM NaCl, 0.34 M Sucrose, 0.2 mM EDTA, 0.2 mM EGTA, 15mMTris-HCl pH7.4, 0.15 mM Spermine, 0.5 mM Spermidine, with one tablet from the “Complete kit” (Roche, Bazel, Switzerland) to avoid DNA damage). The sample was then centrifuged at 1,432xg for 15 min at 4°C, the supernatant discarded and the pellet re-suspended in 200 μL of Buffer I + 1% (v/v) TritonX-100 to remove disrupted cell membranes. The sample was centrifuged again at 1,432xg for 15 min at 4°C and the supernatant was discarded. 200 μL of Buffer I was added to eliminate Triton and maintain DNA integrity. The solution was finally centrifuged at 1,432xg for 15 min at 4°C, the supernatant discarded and the pellet re-suspended in 100 μL of Phosphate Buffered Saline (137 mM NaCl, 2.7 mM KCl, 10 mM Na_2_HPO_4_, 1.76 mM KH_2_PO_4_), with 1 μL of RNaseA (10 μg/mL) to eliminate RNA to avoid cross fluorescence. Finally, the preparation was stored on ice to maintain a monodispersed suspension of cells.

### Characterization of nucleus-stained trophozoites by flow cytometry

**Figure 1:**
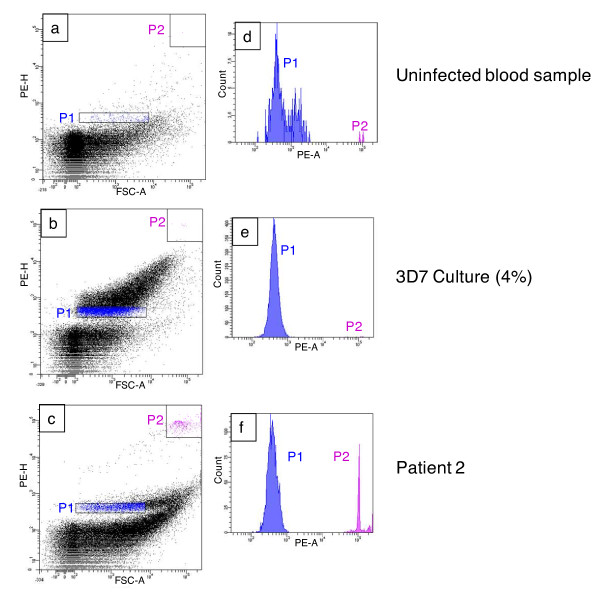
**Isolation of*Plasmodium falciparum*trophozoites by flow cytometry.** On the left panel, Figures 1a, 1b, and 1c represent a two-dimensional scattergram which corresponds to low-angle light scatter (FSC-A) as a function of fluorescence intensity (PhycoErythrine, PE-H). On the right panel, Figures 1d, 1e and 1f represent the number of trophozoites detected (counts) as a function of fluorescence intensity (PE-A). Logarithmic red fluorescence was detected through a 585/42 nm band pass filter (FL2). The speed of sorting corresponded to 5,000 events/sec and for each sample, 50,000 cells were acquired, stored and analysed. Note that the scales of these figures are not the same. P1 window (blue) represents the trophozoite population (the negative control contains debris). P2 window (pink) indicates the position of white blood cells. Figure 1a shows an uninfected blood sample used as a negative control. The corresponding histogram (Figure 1d) shows the distribution of debris. In the middle panel (Figure 1b) the scattergram of the population of parasite stages found in a *P. falciparum* 3D7 culture is shown (Figure 1b). The corresponding histogram (Figure 1e) shows the distribution of trophozoites in the P1 zone. The lower panels (Figure 1c and Figure 1f) represent the parasitaemia of an infected field sample (patient 2 – Table 1).

DNA was stained with Propidium Iodide (PI) at a 10 μg/mL final concentration [14] in a 1.5 mL microtube and kept for at least 1 h in the dark. The stained samples were then analysed with a FACSAria (Becton-Dickinson, Mountain View, CA). The PI-stained nuclei were excited with a 488 nm light and were gated on the basis of their fluorescence intensity (FI) and forward scatter (FSC) [17]. Logarithmic red fluorescence was detected through a 585/42 nm band pass filter (FL2). The speed of sorting was 5,000 events/sec and for each sample, 50,000 cells were acquired, stored and analysed using the FACSDiva software (Becton-Dickinson, Mountain View, CA). The trophozoite area was defined in a two-dimensional scattergram of PE-H (PhycoErythrin-H), which corresponds to fluorescence intensity and FSC (Forward Scatter) primarily by comparing scattergrams for parasite-positive and parasite-negative blood samples (Figure 1). The relative position of the trophozoites and the human cells was determined by comparing cultured parasites with an uninfected blood sample.

### Isolation of trophozoites using an automatic cell sorter device

Trophozoites were then sorted using an Automatic Cell Deposition Unit (ACDU) and placed in standard 96-well plates. Cell sorting was done for 1 and 200 trophozoites per well. Our sorting mode was optimized for maximal purity to ensure that only one target event would be sorted. Each well contained 9 μL of the sample buffer, which was subsequently used for whole-genome amplification (see below). During the entire process, trophozoite solutions were always kept on ice.

### Trophozoite whole genome amplification

Whole genome amplification was carried out using the “Illustra™ GenomiPhi™ V2 DNA Amplification Kit” (GE Healthcare, Uppsala, Sweden) following the manufacturer’s instructions. The trophozoite preparation was denatured by heating at 95°C for 3 min, and then kept on ice. 9 μL of Reaction Buffer (Illustra™ GenomiPhi™ V2 DNA Amplification Kit” - GE Healthcare, Uppsala, Sweden) and 1 μL of enzyme Phi 29 was added, and the preparation incubated at 30°C for 2 h for genome amplification. The amplification was stopped by placing the samples at 65°C for 10 min, before being stored at −20°C.

### Applications

#### Single cell microsatellite genotyping

Single trophozoite whole genome amplified DNA was used as a template for PCR-based amplification of seven polymorphic microsatellites loci (POLYa, TA60, ARA2, Pfg377, PfPK2, TA87, TA109) distributed on five of the 14 *Plasmodium falciparum* chromosomes as listed in Anderson *et al.*[4,5]. A two-step semi-nested PCR strategy with fluorescent end-labelled primers was used for the microsatellite amplification, as described in Razakandrainibe *et al.*[18], following the methodology developed by Anderson *et al.*[4]. The amplified microsatellite repeats were resolved and sized relative to an internal size standard using a Genetic Analyzer 3130xl and the GeneMapper software (Applied Biosystems, Carlsbad, CA). As a positive control, total DNA was genotyped. Each DNA sample was isolated and purified using the DNeasy blood and tissue kit® (Qiagen, Hilden, Germany) according to the manufacturer’s instructions.

To perform this study a total of seven blood samples collected from six infected patients in Senegal (see Table 1, Patients 2–7) and one from The Republic of the Congo (Table 1, Patient 1) were used. As a positive control, an *in vitro* culture of *P. falciparum* trophozoites (strain 3D7) was used. A non-infected blood sample was also used as a negative control. For each sample, 10 single trophozoites were sorted and genotyped.

**Table 1:** Mean percentage of amplified loci overall 7 microsatellite loci for trophozoites and parasitaemia per sample (NA = Not Available)

**Sample name**	**1 trophozoite**	**Parasitaemia**
3D7 culture	30%	NA
Patient 1	57%	4.9. 10^4^
Patient 2	66%	7.6. 10^3^
Patient 3	54%	1.7. 10^6^
Patient 4	21%	5.1. 10^5^
Patient 5	14%	1.1. 10^6^
Patient 6	8%	1.4. 10^5^
Patient 7	54%	4.8.10^5^
Patient 8	NA	4.2. 10^3^
**Average**	**38%**	**NA**

Real time PCR was used to determine the parasitaemia of each sample following the protocol described by Bourgeois and colleagues [19,20].

#### *Plasmodium falciparum* DNA enrichment and whole genome sequencing

The infected venous blood of one patient from Senegal (patient 8, Table1) was used. Parasitaemia was estimated by Real Time quantitative PCR to be around 4,200 parasites/μL. Human blood contains between 5,000 to 7,000 diploid white nucleated cells per μl, and the haploid human genome is approximately three billion base pairs and that of *P. falciparum* around 23 million base pairs. Accordingly we estimate human DNA to be between 310 and 434 times more prevalent in the total DNA extract than *Plasmodium* DNA.

To enrich the *Plasmodium* DNA preparation, 200 trophozoites were twice sorted from the blood sample (following the protocol described above) and whole genome amplification separately performed on these two sets of trophozoites. The resulting amplified DNA from these replicate genome amplifications were pooled for subsequent sequencing to limit potential misrepresentation of certain parts of the genomes due to a random, and not locus-specific, failure of amplification after WGA.

Whole genome sequencing was performed by Integragen (Evry, France) using a Genome Analyser GAII Illumina machine. Two libraries of fragmented DNA were prepared (average size of fragments: 330 bp) and 36 bases sequenced on both extremities. Sequencing was performed in two lanes, the first with the standard enzyme from the Illumina kit and the second with Herculase II Fusion (Agilent, Santa Clara, CA), an enzyme that reduces the amplification bias due to the AT-rich composition of the *P. falciparum* genome (~ 80% AT). Sequence data were analysed using the Illumina Pipeline and were performed by Integragen.

## Results

### Isolation and trophozoite cell-sorting

To isolate single trophozoites after blood sample extraction, the trophozoite population was characterized using two representative two-dimensional scattergrams. The scattergrams in Figures 1a, 1b and 1c represent fluorescence intensity (PE-H, PhycoErythrin- H) as a function of the forward low-angle light scatter (FSC-A), and the scattergrams in Figures 1d, 1e and 1f represent the distribution of the number of sorted cells (counts) as a function of PE-A (Phycoerythrin-A), which corresponds to propidium iodide fluorescence intensity. Figures 1a, 1b and 1c show different cell populations in venous blood samples after trophozoite extraction: ring stage trophozoite populations (P1, blue), nucleated white blood cell populations (P2, pink) and debris stained with Propidium Iodide. Each dot is considered as one event defined as trophozoite, white blood cell or debris. Debris may have different origins: damaged or fragmented host nuclei, aggregates of membranes where free DNA is fixed. Thus the presence of DNA causes their fluoresce and can be misdiagnosed as trophozoite nuclei (i.e. if present in the P1 zone). In addition to that, debris may correspond to background noise caused by the machine. This is more likely as we worked at the logarithmic scale to increase detection sensitivity (due to the small amount of DNA within trophozoite nuclei). For each treated sample; trophozoites were always sorted from the P1 window and isolated for genetics studies.

### Single-cell genotyping

Single trophozoites were amplified at seven microsatellite loci. The average rates of amplification for each sample are shown in Table 1. On average, amplification success was 38%. Strong variations in amplification success were however observed among single-trophozoite samples (8% to 66% microsatellite amplification success).

### DNA enrichment and whole genome sequencing

Two lanes of sequences were generated for each sample. One lane using the classic Illumina enzyme, and another the Herculase II Fusion enzyme, which reduces the amplification bias of AT-rich genomes such as that of *P. falciparum*. 12,455,400 clusters (passing filters) were obtained for the first lane and 9,713,500 for the second one. The percentage alignment to the *Plasmodium* reference genome was higher for the second lane probably due to higher amplification of the *Plasmodium* genome using the Herculase enzyme.

Despite an estimated initial ratio of human to *Plasmodium* DNA ranging from 310 to 434 to 1, the method gave a final DNA sample composed on average by 62% *Plasmodium* DNA, 20% human DNA and 18% DNA that did not blast with any human or *Plasmodium* reference genomes (Table 2). Using this method, the enrichment level, calculated as the initial human/*Plasmodium* DNA ratio over the final human/*Plasmodium* DNA ratio was between 939 and 1283.

**Table 2:** **Number of clusters passing filters obtained and percentage aligning on the*Plasmodium falciparum*(3D7 strain) reference genome and on the human reference genome (Hg18)**

**Enzyme used**	**Number of passing clusters**	**% of passing clusters**	**% aligning on the*P. falciparum*reference genome (3D7)**	**% aligning on the human reference genome (Hg18)**	**% foreign DNA**
Illumina	12 455 400	92.00	55.53	20.41	24.06
Herculase	9 713 500	93.00	69.45	21.12	9.43
**Average**	**11 084 450**	**92.5**	**62.49**	**20.77**	**16.74**

Regarding the quality of the *P. falciparum* genome sequence produced, reads obtained across all lanes covered 99% of the genome and were used to assemble 80% of the *P. falciparum* genome*.* The average coverage was 43X.

## Discussion

The aim of this paper was to test two possible applications of flow cytometry and cell sorting for malaria genetic studies: (1) single trophozoite genotyping and (2) trophozoite DNA enrichment for subsequent whole genome sequencing. The idea of the paper was to use protocols already described and published and to combine them to test for these two applications. It is important to note that the method presented here can certainly be improved for better yields. Note moreover that although the current protocol was optimized for sorting *P. falciparum* ring trophozoites, the flow cytometric parameters for sorting other parasite stages (schizonts, gametocytes) [21] or other species could be determined.

### Single cell genotyping

It was demonstrated, for the first time, the possibility to genotype single trophozoites isolated from frozen clinical blood samples using different microsatellite markers. The average proportion of alleles that were successfully amplified from single trophozoites was 38%, but this varied strongly between samples (from 8% to 66%). Several factors may explain these variations. First, some trophozoites might not have been loaded properly into the wells during sorting. Second, this could be due to improper sorting itself [9]. Indeed, as previously noted, the trophozoite area (P1) in which cells were picked during flow cytometry and sorting comprises both trophozoites and debris. Consequently, debris can also be sorted, which results in no amplification. Finally, this could be due to issues related to the conservation of blood samples and DNA. Indeed, all samples were conserved frozen for a long time (many for more than 10 years) and have undergone recurrent freeze-thawing cycles which may have led to DNA degradation [22].

As observed by Frumkin *et al.*[23], it is likely that the amplification success rate would be higher with single cells extracted from fresh samples. In the case of malaria research, methods of sorting have already been developed to specifically deal with these kinds of samples (cultivated or short terms cultivated parasites) [9,14,24,25]. These methods could, in principle, also be adapted to perform single cell genotyping. This is particularly true for the method developed by Miao *et al.*[9] that allows the rapid isolation of single malaria parasite-infected red blood cells by cell sorting.

Single cell genotyping opens new possibilities for the characterisation of the different haplotypes present within mixed-infections. Although the amplification success obtained here is too low to allow proper determination of each haplotype, the technologies used in genetics for genotyping or sequencing are improving so rapidly it is likely that better yields could be obtained soon. The limits of the method for archival frozen samples will certainly be fixed by the level of degradation of DNA due to recurrent freeze-thaw cycles.

### *Plasmodium* DNA enrichment and whole genome sequencing

A major challenge to sequencing clinical malaria samples using next generation sequencing is the abundance of “contaminating” human DNA. Removal of leukocytes and other components from infected blood samples is, therefore, an important prerequisite.

Methods have recently been proposed to deplete white blood cells from the blood of infected patients [10,26] for subsequent whole genome sequencing of *Plasmodium* isolates. But these methods only deal with fresh samples. The only method that currently deals with frozen samples is the method developed by Melnikov *et al.*[11] that purifies parasite DNA using hybrid selection, but this method requires a good knowledge of the target genome.

The method described provides a good alternative as it does not rely on particular knowledge of the target genome and can, in principle, be adapted to any parasite species as long as it is possible to distinguish it from the host nucleated blood cells. Especially since, the enrichment level obtained in this trial was very similar to that obtained using other current existing methods [10,11]. Thus; from an initial blood sample estimated to present a ratio of 1 *Plasmodium* nucleotide to 310–434 human nucleotides, we finally obtained a DNA product composed of ~ 3 *Plasmodium* nucleotides to every human one. Moreover, the quality of the assembled genome was high covering around 80% of the reference *P. falciparum* genome (3D7 strain) with a depth of about 43X.

Although promising, a complete test of the method is required to assess its sensitivity and yields using samples with different parasite loads.

## Conclusions

To conclude, this method allows the isolation, genotyping and sequencing of *P. falciparum* trophozoites from archival clinical blood samples. A lot of these precious samples, stored in freezers all over the world, are currently overlooked for population genetic studies because of technical limitations (multiclonal infections and host DNA contamination). This method can easily be adapted to other pathogens or other malaria blood stages (e.g. gametocytes). In addition, the fact that our method applies to frozen samples makes it usable in most endemic settings. Once blood samples have been collected, it is indeed easy to freeze them until processed. The development of such methods is important as it opens new avenues for the understanding of the dynamics of multiple infections *in natura* as well as their role for parasite evolution.

## Competing interests

The authors declare that they have no competing interests.

## Authors’ contributions

AB, CA, PD, FR, FP designed the study. AB, CA, CD, PD, LL, LB performed the experiments. AB, CA, PD, CD, FR, FP analysed the data. All authors put forward different ideas and contributed to the early draft. AB, CA, FP wrote the paper. All authors agreed the final draft. *AB and CA contributed equally to this work. All authors read and approved the final manuscript.
